# Unravelling the sex- and age-specific impact of poaching mortality with multievent modeling

**DOI:** 10.1186/s12983-019-0321-1

**Published:** 2019-06-13

**Authors:** Luca Corlatti, Ana Sanz-Aguilar, Giacomo Tavecchia, Alessandro Gugiatti, Luca Pedrotti

**Affiliations:** 1 Chair of Wildlife Ecology and Management, University of Freiburg, Tennenbacher Straße 4, 79106 Freiburg, Germany; 2 Stelvio National Park, Via De Simoni 42, 23032 Bormio, Italy; 3 Freiburg Institute for Advanced Studies, University of Freiburg, Albertstraße 19, 79104 Freiburg, Germany; 4 Animal Demography and Ecology Unit, Instituto Mediterráneo de Estudios Avanzados, IMEDEA (CSIC-UIB), Esporles, Islas Baleares Spain; 5 Applied Zoology and Animal Conservation Group, University of Balearic Islands, Palma, Spain

**Keywords:** Capture-recapture, Deer, GPS, Illegal hunt, Multievent models, Recovery, Resighting

## Abstract

**Background:**

Poaching is a prominent source of ‘hidden hurdles’, cryptic impacts of human activities that may hinder the conservation of animal populations. Estimating poaching mortality is challenging, as the evidence for illegal killing is not outwardly obvious. Using resighting and recovery data collected on 141 marked red deer *Cervus elaphus* within the Stelvio National Park (central Italian Alps), we show how multievent models allow to assess the direct impacts of illegal harvesting on age- and sex-specific survival, accounting for uncertainty over mortality causes.

**Results:**

Mortality caused by poaching was consistently higher for males than for females in all age classes. In males, the probability of dying from poaching was higher for extreme age classes, while in females all age classes showed fairly similar values of poaching mortality. The strong bias in sex-specific poaching mortality was possibly due to trophy killing in adult males and ‘bushmeat-like’ killing for private or commercial gain in young males and in females.

**Conclusions:**

A robust assessment of age- and sex-specific prevalence of poaching in wildlife populations is pivotal when illegal killing is of conservation concern. This provides timely information on what segment of the population is most likely to be affected. Besides obvious demographic consequences on small populations, age- and sex-biased poaching prevalence may contrast with the need to maintain ecosystem complexity and may alter behavioral responses to human presence. The information provided by multievent models, whose flexibility makes them adaptable to many systems where individual-based data is part of population monitoring, offers a support to design appropriate strategies for the conservation of wildlife populations.

**Electronic supplementary material:**

The online version of this article (10.1186/s12983-019-0321-1) contains supplementary material, which is available to authorized users.

## Background

Knowing the impacts of human activities on animal behavior [1], population structure [2] and, ultimately, population dynamics, is pivotal to design management strategies for the conservation of wildlife populations [3]. Through this knowledge, conservation management aims to secure ecosystem complexity, while allowing for the sustainable use of wildlife – be it for consumptive or non-consumptive purposes.

Some impacts, however, are difficult to either predict, study or quantify. Recreational activities or trophy hunting, for example, may have negativeimpacts on animals’ vigilance activity and personality traits, e.g., in elk *Cervus canadensis* [4, 5], population genetic quality, e.g., in bighorn sheep *Ovis canadensis* [6] or, more generally, social structure in ungulates [7]. Despite their potential negative effects on wild populations, such ‘hidden hurdles’ remain hard to integrate into decision-making protocols. One of the most prominent human-related sources of ‘hidden hurdles’ in wildlife conservation is poaching, an illegal and lethal activity whose direct and indirect impacts are, by their very nature, cryptic [8].

For critically endangered mammals, the relevance of illegal killing is obvious from a demographic standpoint (e.g., rhinos [9] or Amur tiger *Panthera tigris altaica* [10]). Poaching, however, may have less predictable consequences, including for example the disruption of social structures and of mating system [11]. Poaching consequences may be relevant also in species of less conservation concern. For instance, in the European roe deer *Capreolus capreolus* illegal killing can undo the effects of planned management strategies, possibly jeopardizing the sustainable use of resources [12]. Poaching on common species can also have negative effects on ecosystem complexity and food webs, as poachers may compete with large carnivores over food resources and threaten their survival [13]. These effects may be expected to aggravate when legal and illegal killing co-occur, in absence of compensatory mortality [14]. The assessment of age- and sex-specific mortalities associated with poaching provides timely information on the sensitivity of individuals to illegal harvesting. This, in turn, may help to design appropriate management strategies where poaching is a concern. Nonetheless, estimating poaching-related mortality is challenging [8].

Unbiased estimates of the prevalence of illegal hunting may be obtained through long term monitoring of marked animals and analysis with capture–mark–recapture (CMR) models to account for detection failure, e.g. [15, 16]. To date, however, the models proposed to estimate poaching mortality seldom included tag loss / GPS malfunction (but see [17]) and never accounted for uncertainty over the causes of death. Multievent CMR models are particularly suited for estimating mortality probabilities by different sources [18] while accounting for changes in mark type carried by the individuals [19, 20], different detection probability among marks, as well as for uncertainty in the causes of death of recovered carcasses.

We used a multievent CMR modeling approach [18] to model age- and sex-specific poaching prevalence in red deer *Cervus elaphus* within a protected area in the Italian Alps using data collected on differently marked individuals. The red deer is widely distributed across Europe, albeit patchily [21], but some *taxa* are of conservation concern (e.g., Corsican red deer *Cervus elaphus corsicanus*, Mesola deer *Cervus elaphus italicus*, barbary deer *Cervus elaphus barbarus*) [22]. The assessment of the impact of poaching mortality may thus provide timely information to maintain viable populations for, e.g., ecosystem complexity, contemplation by tourists, education or research where the species is abundant, and to improve conservation status where poaching is a threat (cf. [23] for Corsican red deer).

Our analytical framework for the estimation of poaching mortality incorporates tag loss, detection failure, heterogeneity of recapture probability and uncertainty over mortality causes. We show how model flexibility can be used to provide robust estimates of the direct impact of poaching on age- and sex-specific mortality probabilities, relative to other causes of death. Given the cryptic nature of poaching, no a priori hypotheses could be formulated on the age- and sex- specific impacts of illegal killing in our study population.

## Methods

### Study site and data collection

**Fig. 1:**
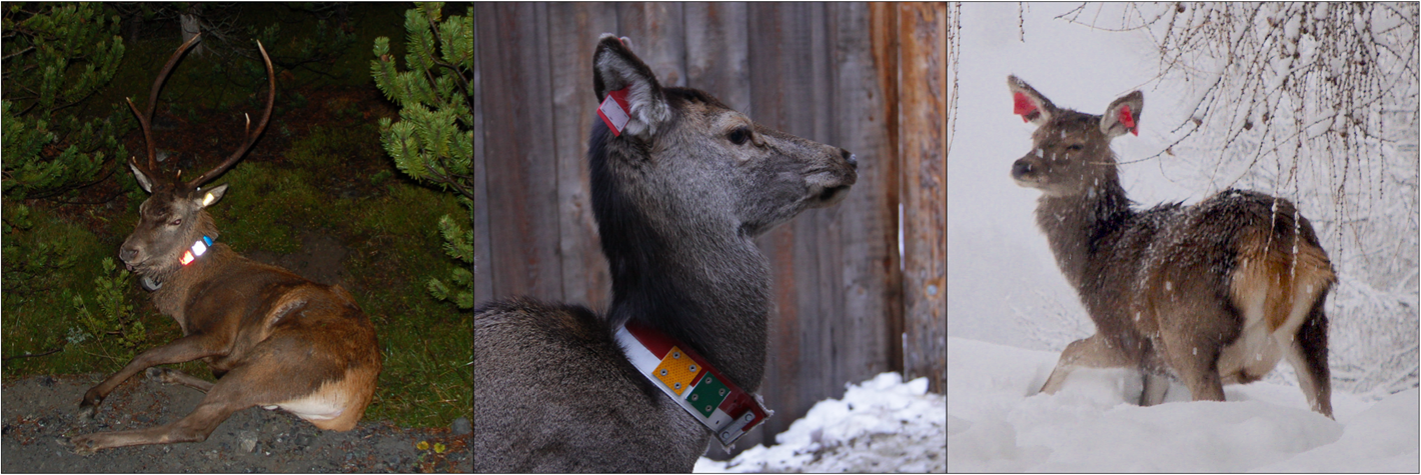
Adult male with GPS collar and ear tags (left), adult female with optical collar and ear tags (centre) and young male with ear tags only (right). All tags were equipped with colored reflectors to facilitate individual recognition during spring spotlight counts, as exemplified by the male on the left. All pictures were taken during live captures

The study was conducted from 2007 to 2017 in an area of ca. 27,900 ha between 1200 and 3850 m a.s.l. within the Stelvio National Park, central Italian Alps (10°25′N, 46°27′E). Here, red deer abundance increased sharply over the past decades, reaching current densities of about 27.4 ind./km^2^ (± 2.5 SD), cf. [24]. In 2011 the Park Authority established a culling plan to limit deer impacts on forest regeneration and agricultural activities. Culling always occurred between late October and early February, with the support of professional hunters under the supervision of the Park Authority. As the aim was to reduce deer density, the sex-ratio in the culling plan was biased towards females (2:1); no restrictions were imposed on females in terms of age and lactating status, whereas only males < 6 years of age could be culled. Marked deer were not allowed for culling. During the study period 56 males and 85 females were captured by darting between October and April. After sedation, each animal was assigned to a given sex and age, based on inspection of tooth wear and eruption. All 141 individuals were marked with ear tags; 35 males and 83 females were additionally marked with colored belts with unique pattern of colors and reflectors (hereafter ‘optical collars’). Twenty-nine of these animals (13 males and 16 females) had a Global Positioning System (GPS, Vectronic Aerospace GmbH) mounted on the optical collar. In summary, animals were of three ‘mark types’: ear tags only, ear tags plus optical collars, ear tags plus optical collars equipped with a GPS device (Fig. 1). All GPS collars had a timer-controlled drop-off device set to trigger after 3 years. Therefore, GPS-collared individuals were allowed to become ‘plainly ear-tagged deer’ (in case of successful drop-off of the collar) or ‘optical-collared deer’ (in case of drop-off failure and cease of the GPS signal). Individuals with a working GPS device had perfect detection probabilities, as GPS locations were automatically collected on an hourly basis until the battery ran out, the GPS failed permanently, or until the collar dropped-off. Resightings of deer with inactive or dropped-off GPS collars, optical collars and/or ear tags only were conducted using spotlight counts between mid-April and mid-May (details in [24]).

### Data coding, model definition and selection

Live encounter data were collected from mid-April to mid-May of each year between 2008 and 2017 and coded into individual encounter histories. Observations were stratified according to the mark-type. We assigned ‘1’ to individuals detected with working GPS devices; ‘2’ to individuals observed alive with optical collar; ‘3’ to individuals observed with ear tags; ‘4’ to individuals resighted with inactive GPS collars (see Additional file 1 for further details on observation coding). Animals alive with inactive GPS collar have a detection probability similar to those with optical collars, but this code had to be specified to account for drop-off failure. A ‘0’ was assigned to individuals not observed on a given year. In addition to live encounter data, dead recoveries (*n* = 50; 20 males and 30 females) were reported by the park personnel or by local people and tourists on a non-systematic basis. Deer carcasses found in the field were subsequently inspected by a veterinarian, who established the likely causes of death, thereby minimizing the uncertainty associated to death cause assignment. Causes of death were defined as: ‘poaching’ (*n* = 16 individuals found within the park boundaries in no-hunting periods, with gunshot wounds or in abnormal situations, e.g., buried under snow), ‘others’ (*n* = 25, e.g., starvation, collision with cars or legally shot outside of the Park boundaries) and ‘unknown’ (*n* = 9 deer for which causes of death could not be unequivocally assigned). Dead encounters were coded depending on mark type and cause of death: ‘5’ (poached, with working GPS); ‘6’ (poached, with inactive or dropped-off GPS, optical collar or ear tags); ‘7’ (dead by other causes, with working GPS); ‘8’ (dead by other causes, with inactive or dropped-off GPS, optical collar or ear tags); ‘9’ (unknown causes, with working GPS); ‘10’ (unknown causes, with inactive or dropped-off GPS, optical collar or ear tags; cf. Additional file 1). Recovery data for individuals with optical collars (*n* = 29) and ear tags only (*n* = 6) were pooled to avoid identifiability problems caused by the small sample size for the latter mark type.

The model for the encounter histories described above was specified using a multievent statistical framework [18]. As no goodness-of-fit (GOF) test is available for multievent models, we first tested the adequacy of a model assuming survival and resight parameters as time-dependent (i.e., the Cormack-Jolly-Seber model), using live observations only (codes 2, 3, and 4) in program U-CARE 2.3.2 [25]. Observations collected by GPS-transmitters and recoveries were not considered for GOF because the former have perfect probability of recapture and the latter were too sparse for full time-dependent models. In the following paragraph we provide a verbal description of our multievent modeling procedure, technical details are reported in Additional file 1.

We specified the multievent models with three sets of parameters: 1) the initial state probabilities, i.e., the initial proportions of individuals with different mark type; 2) the probabilities of transition between the states, decomposed in the probabilities of different types of mark loss and the probability of mortality; 3) the probabilities of resight and recovery (Additional file 2). We considered an additional parameter to accommodate the uncertainty over the cause of death, so that the unknown causes were automatically ‘redistributed’ between the known ones. Due to identifiability issues, this parameter was modelled to independent of the cause of death. The model included 9 different states representing the type and status of the mark (working GPS collar, inactive GPS collar, optical collar, ear tags), the state alive or dead, and the cause of mortality (poaching vs. others) and 11 possible events (see above). Parameters were estimated simultaneously by maximum likelihood with program E-SURGE [26]. We applied the same model design in males and females, separately.

Model selection began by fitting a general model for either sex, assuming the following structure: two age classes for the parameter ‘GPS-signal loss’ (GPS collars were set for a 2 year duration), a constant probability of GPS-transmitter drop-off, an age effect in survival using different age structures for young, adult and old animals based on the existing literature, e.g. [2, 27, 28], time- and mark type-dependent resighting probabilities, time-dependent recovery probabilities and a constant probability to determine the cause of death. We first selected the age structure that minimized the value of Akaike’s Information Criterion adjusted for the effective sample size (AICc [29]). We used this structure to test for time dependence in the recovery probability, and subsequently assess the effects of time and type of mark on resighting probabilities. Finally, we modelled different sources of mortality across age classes, in either sex, by contrasting 3 alternative scenarios: 1) mortalities vary interactively with age; 2) mortalities vary in parallel with age (additive model); 3) mortalities vary independently with age (constant model). Model selection was based on AICc values. Akaike’s weight (*w*_*j*_) for each model *j* in the final candidate set was calculated as an index of relative model plausibility and used to obtain averaged estimates [29].

## Results

The goodness-of-fit test of the Cormack-Jolly-Seber time-dependent model – with only live observations – indicated an adequate description of the data (χ^2^ = 45.86, df = 47, *p* = 0.52). The modeling of age effects on female mortality probabilities (Models 1F–3F, Table 1) supported different survival probabilities between yearlings (1 year), adults (2–7 years) and old animals (8+ years) (Model 1F, Table 1). For males (Models 1 M–3 M, Table 1) a slightly different age structure proved a better fit to the data: young (1–3 years), adults (4–7 years) and old animals (8+ years) (Model 2 M, Table 1). Models with constant recovery probabilities were selected in both sexes (Models 4F and 4 M, Table 1) whereas resighting probabilities showed temporal variation in females (Model 6F, Table 1) and varied depending on mark type in males (Model 7 M, Table 1). In females, the model with the lowest AICc value indicated that poaching mortality was constant across all age classes (Model 10F, Table 1). This model had a similar AICc value to the model assuming parallel variation of mortality causes with age (Model 9F, Table 1). In males, the age-dependent model with additive effects of mortality causes returned the lowest AICc value (Models 9 M, Table 1).

**Table 1:** Models considered to investigate mortality by poaching and other causes, resighting and recovery probabilities of female (F) and male (M) deer within the Stelvio National Park, between 2008 and 2017. The table reports: model structure for mortality, resight and recovery probability; *np* number of parameters, *Dev* relative deviance, *AICc* Akaike Information Criterion corrected for sample size, *ΔAICc* the AICc difference between the current model and the model with the lowest AICc value; *w* = Akaike’s weight calculated for the candidate models (i.e. models fitted to investigate biological hypotheses on survival); Hypothesis: different hypotheses on the impact of poaching relative to other causes of mortality. Model notation: in ‘A’, numbers represent the age intervals; ‘t’ = temporal effects; ‘×’ and ‘+’ indicate interactive and additive effect, respectively; ‘,’ = different parameters were considered; ‘.’ = constant; ‘Poach’ = poaching; ‘Tag’ = ear tags; ‘Collar’ = optical collars and inactive GPS collars. All models assumed: a 2-year variation in the probabilities of losing GPS signal after deployment; constant probability of GPS drop-off; constant probabilities of determining causes of death. Hypothesis-driven models are in italics, models with the lowest AICc for either sex are in bold

Model	Mortality	Resight	Recovery	np	Dev	AICc	ΔAICc	*w*	Hypothesis
1 F	[Poach, Other] × A(1,2:7,8+)	[Tag, Collar] × t	t	40	820.13	910.51	28.79		
2 F	[Poach, Other] × A(1:3,4:7,8+)	[Tag, Collar] × t	t	40	821.42	911.80	30.08		
3 F	[Poach, Other] × A(1,2:3,4:7,8+)	[Tag, Collar] × t	t	42	819.68	915.19	33.47		
4 F	[Poach, Other] × A(1,2:7,8+)	[Tag, Collar] × t	.	31	826.27	894.38	12.66		
5 F	[Poach, Other] × A(1,2:7,8+)	[Tag, Collar] + t	.	24	835.38	887.00	5.28		
*6 F*	*[Poach, Other] × A(1,2:7,8+)*	*t*	*.*	*23*	*835.38*	*884.70*	*2.98*	*0.106*	*Mortalities vary interactively with age*
7 F	[Poach, Other] × A(1,2:7,8+)	[Tag, Collar]	.	15	858.43	889.84	8.12		
8 F	[Poach, Other] × A(1,2:7,8+)	.	.	14	858.45	887.67	5.95		
*9 F*	*[Poach, Other] + A(1,2:7,8+)*	*t*	*.*	*21*	*837.15*	*881.91*	*0.19*	*0.426*	*Mortalities vary in parallel with age*
***10 F***	***[Poach]*** **vs** ***Other × A(1,2:7,8+)***	***t***	***.***	***21***	***836.96***	***881.72***	***0.00***	***0.469***	***Poaching mortality is constant***
1 M	[Poach, Other] × A(1,2:7,8+)	[Tag, Collar] × t	t	39	498.68	607.88	45.75		
2 M	[Poach, Other] × A(1:3,4:7,8+)	[Tag, Collar] × t	t	39	492.34	601.54	39.41		
3 M	[Poach, Other] × A(1,2:3,4:7,8+)	[Tag, Collar] × t	t	41	492.14	609.29	47.16		
4 M	[Poach, Other] × A(1:3,4:7,8+)	[Tag, Collar] × t	.	31	503.03	583.40	21.27		
5 M	[Poach, Other] × A(1:3,4:7,8+)	[Tag, Collar] + t	.	24	510.54	568.97	6.85		
6 M	[Poach, Other] × A(1:3,4:7,8+)	t	.	23	514.66	570.18	8.05		
*7 M*	*[Poach, Other] × A(1:3,4:7,8+)*	*[Tag, Collar]*	*.*	*15*	*530.44*	*564.31*	*2.18*	*0.251*	*Mortalities vary interactively with age*
8 M	[Poach, Other] × A(1:3,4:7,8+)	.	.	14	536.32	567.68	5.55		
***9 M***	***[Poach, Other] + A(1:3,4:7,8+)***	***[Tag, Collar]***	***.***	***13***	***533.24***	***562.13***	***0.00***	***0.744***	***Mortalities vary in parallel with age***
*10 M*	*[Poach]* vs *Other × A(1:3,4:7,8+)*	*[Tag, Collar]*	*.*	*13*	*543.16*	*572.04*	*9.91*	*0.005*	*Poaching mortality is constant*

**Fig. 2:**
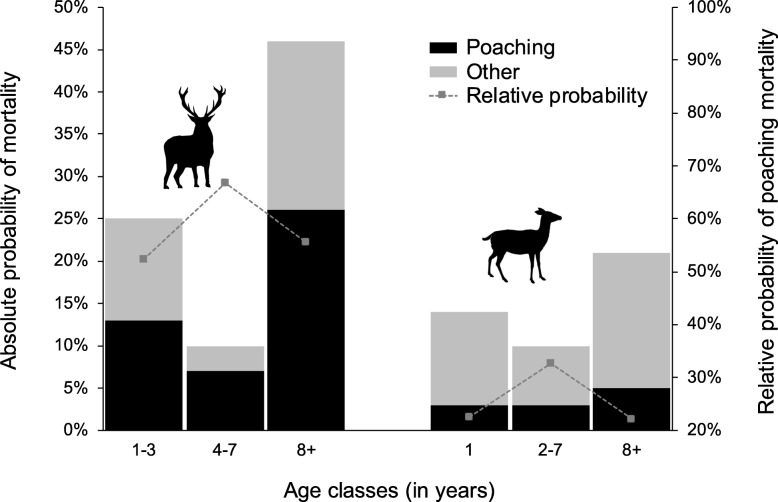
Absolute yearly probability of mortality due to poaching (black bars) and other causes (grey bars) and relative yearly probability of poaching mortality (dashed grey line) for different age classes in male (on the left) and in female (on the right) deer, within the Stelvio National Park, between 2008 and 2017. The total probability of mortality is given by the sum of poaching (black bars) and other causes (grey bars)

Averaged estimates indicated that GPS-transmitters began to fail the second year after deployment: the probability of signal loss was slightly higher for females (0.39, 95%CI = 0.17–0.66) than for males (0.27, 95%CI = 0.07–0.64). Annual probability of GPS drop-off was 0 for females and 0.15 for males (95%CI = 0.05–0.39). Dead recovery probabilities were 0.47 (95%CI = 0.33–0.61) for females and 0.38 (95%CI = 0.24–0.55) for males. Female resighting probabilities varied over time between 0.26 and 0.96 (mean = 0.75) and did not depend on the type of mark. Males marked with optical collars showed higher resighting probabilities (0.47, 95%CI = 0.33–0.61) than males marked with ear tags only (0.23, 95%CI = 0.13–0.36). Averaged survival estimates in males and females showed age-dependent variations, with highest values for the intermediate classes (Table 2). However, while females had fairly consistent survival probabilities across ages, males showed much greater fluctuations (Table 2). The estimates of mortality caused by poaching were consistently higher for males than for females in all age classes (Table 2, Fig. 2). In males, the probability of dying from poaching was higher for young and – especially – old individuals while in females all age classes showed fairly similar values of poaching mortality (Table 2, Fig. 2). Notably, in males the point estimates of poaching mortality were consistently higher than those of other mortality events, accounting for 52% of the total mortality in young individuals, 67% in adults and 56% in old deer (Fig. 2). In females, poaching accounted for *c*. 22% of the total mortality in yearling, 33% in adult hinds, and 22% in old individuals (Fig. 2).

**Table 2:** Averaged estimates of survival and poaching mortality probabilities for marked deer within the Stelvio National Park, between 2008 and 2017. Poaching mortality estimates refer to the absolute probability that an individual alive at year t-1 would be poached at t. The table reports estimates, standard errors (SE) and 95% lower and upper confidence level for different age classes in either sex

Parameter	Sex	Age	Estimate	SE	95% LCL	95% UCL
Survival	Males	1–3	0.753	0.063	0.612	0.855
		4–7	0.902	0.047	0.765	0.963
		8+	0.541	0.100	0.348	0.723
	Females	1	0.862	0.078	0.631	0.957
		2–7	0.898	0.023	0.844	0.935
		8+	0.794	0.033	0.722	0.851
Poaching mortality	Males	1–3	0.129	0.047	0.062	0.255
		4–7	0.066	0.033	0.024	0.168
		8+	0.255	0.082	0.129	0.443
	Females	1	0.031	0.017	0.011	0.091
		2–7	0.033	0.013	0.015	0.072
		8+	0.046	0.018	0.021	0.099

## Discussion

Assessing poaching prevalence in wildlife populations is difficult. The multievent capture-mark-recapture modeling approach used here allowed to efficiently estimate sex- and age-specific mortality due to illegal killing, while accounting for different types of marks, tag loss and uncertainty over the causes of death. This information is of paramount importance for appropriate conservation management strategies, as it allows to identify the segments of the population most affected by poaching. Furthermore, the flexibility of multievent modeling makes this approach suitable for any study system where the collection of individual-based data is part of population monitoring (e.g., wolf *Canis lupus* [30]; brown bear *Ursus arctos* [31]).

Poaching-related mortality is among the main issues for the conservation of many threatened species, from large mammals to reptiles (e.g., rhinos [9]; cheetah *Acinonyx jubatus* [32]; leatherback turtles *Dermochelys coriacea* [33]). Less intuitively, poaching may have negative effects also in non-threatened species, when it causes depletion that may affect natural food webs (e.g., in carnivore-ungulate systems [13]) or when it contrasts with management plans aimed at securing a sustainable use of resources for consumptive and non-consumptive purposes. Here we found that male deer suffered a much greater poaching mortality than females in all age classes, with peaks of 25% in old stags (Table 2). In females, poaching accounted for 3–4% of the mortality, regardless of age. Although poaching may not raise immediate conservation concerns in super-abundant species, consequences may be expected to occur at different levels.

Management plans typically aim to achieve multiple goals. In our study site, for example, deer management aimed to lower consequences of deer presence on the ecosystem by reducing negative impacts on forest regeneration and agricultural activities, while conserving a viable population and a fearless behavior in deer, for contemplation by tourists. In this respect, the consequences of poaching are not straightforward to predict. While, in principle, poaching may help reducing browsing impacts by lowering densities in super-abundant ungulates, it may contrast with the long-term interest of maintaining demographically viable populations. From a demographic standpoint, in fact, age- and sex-biased poaching mortality is expected to bear fitness consequences [34]. Our data, for example, show evidence for the occurrence of trophy-killing on adult males. Although there is no evidence of a positive association between breeding values for antler size and fitness in red deer, breeding success correlates positively with antler size [35]. Furthermore, the removal of adult individuals from the population may lead to a greater reproductive investment by young adults, with potential detrimental consequences on their overwinter survival [34]. To ensure the maintenance of viable populations, which are crucial to ecosystem complexity – especially when large predators are present [13], investigations on long-term demographic consequences of the sex- and age-specific impact of illegal killing are warranted, for example through the integrated analysis of surveys and individual-based information on poaching prevalence [31, 36].

Poaching may also bear consequences at the behavioral level, and the investigation of poaching prevalence may help depicting negative effects on wildlife populations. The occurrence of trophy-killing, for example, suggests that poachers may concentrate their activity during the rutting season, when the opportunity for successful poaching on adult males is greatest. This may select for fearful behavioral responses (cf. [5]) and make animals avoid human presence, in contrast with conservation strategies that aim to guarantee the possibility of contemplation by tourists (e.g., during the mating season). Furthermore, the assessment of age- and sex-specific poaching prevalence may provide clues to optimize survey activity by wildlife wardens which, in our case, may concentrate during the rutting period, to avoid undesirable consequences of poaching on males.

## Conclusions

The information provided by multievent models, whose flexibility makes them adaptable to many systems where individual-based data is part of population monitoring, offers a support to design appropriate strategies for the conservation of wildlife populations. It is worth noting that our approach to investigate poaching prevalence can be extended to different systems where individual-based data are available. Monitoring individual-based information in natural populations entails the need to account for detection probability. When multiple marks are considered, there is the additional problem of dealing with a detection probability that depends on mark type and possibly with mark loss. [17] proposed a multievent model based on conditional processes to incorporate tag loss and different type of detection probabilities. Here, we have expanded this model to include a larger number of mark types and, more importantly, the uncertainty over mortality causes. This expansion, which allowed to include recoveries from uncertain causes, has also entailed a new event and a new set of parameters. The trade-off between additional information and model complexity should be carefully evaluated. A similar trade-off exists in including additional age classes or mark types. Despite these limitations, the multievent modelling approach allowed to investigate the age- and sex-specific impacts of poaching relative to other causes of mortality, framing the heterogeneity due to mark type, multiple data sources and uncertainty over mortality causes.

In our study, several deer were equipped with radio-transmitters. Radio-tracking studies have two important limitations: due to the costs of transmitters, only a relatively small number of animals can be marked and the information only lasts the lifespan of the battery. We showed how to integrate this information with those collected by using only ear tags. The current model can be additionally used, for example, to explore through simulations the costs and benefits of different mark methods and provide indications for planning the optimal study design given the available funds (e.g. radio-transmitters vs. ear tags) for species of conservation concern.

## Additional files

Additional file 1: Multievent model design. (PDF 205 kb)

Additional file 2: Multievent model representation. (PDF 163 kb)

Additional file 3: Dataset (.csv) used for analysis. (CSV 4 kb)

## Data Availability

Details about multievent model design and representation are included in Additional files 1 and 2, respectively. The dataset supporting the conclusions of this article is included in Additional file 3.

## Abbreviations

AICc: Akaike Information Criterion corrected for small samples
CMR: Capture-Mark-Recapture
GOF: Goodness Of Fit
GPS: Global Positioning System
SD: Standard Deviation
