# Supplementary material for: Unravelling the sex- and age-specific impact of poaching mortality with multievent modeling
Source: Front Zool. 2019 Jun 13;16:20. doi: 10.1186/s12983-019-0321-1 (PMC6567384; doi:10.1186/s12983-019-0321-1)
Supplement: Supplementary file 1 — Multievent model design. (PDF 205 kb) [file 12983_2019_321_MOESM1_ESM.pdf]

Additional File 1

Multievent model design

The eleven different events considered in our multievent model are:

‘0’ (individual not observed on a particular year)

‘1’ (individual detected alive with a functioning GPS signal)

‘2’ (individual observed alive and identified by means of its optical collar)

‘3’ (individual observed alive and identified by means of its ear tags)

‘4’ (individual resighted alive and identified by means of its GPS collar mark but without an active GPS signal)

‘5’ (individual recovered recently dead with a functioning GPS signal, being its death caused by poaching)

‘6’ (individual recovered recently dead with inactive or dropped-off GPS, optical collar or ear tags, being its death caused by poaching)

‘7’ (individual recovered recently dead with a functioning GPS signal, being its death caused by other causes)

‘8’ (individual recovered recently dead with inactive or dropped-off GPS, optical collar or ear tags, being its death caused by other causes)

‘9’ (individual recovered recently dead with a functioning GPS signal being its cause of death unknown)

‘10’ (individual recovered recently dead with inactive or dropped-off GPS, optical collar or ear tags, being its cause of death unknown).

The model includes 9 underlying biological states. Four states for live individuals coded:

A_{AG} (i.e., alive with an active GPS signal)

A_{FG} (i.e., alive with an inactive GPS signal)

A_{OC} (i.e., alive with an optical collar)

A_{ET} (i.e., alive with an ear tag)

And five states for dead individuals, coded:

DP_G (i.e., recently dead by poaching with an active GPS signal)

DP_{CT} (i.e., recently dead by poaching with an inactive GPS signal, an optical collar or an ear tag)

DO_G (i.e., recently dead by other causes with an active GPS signal)

DO_{CT} (i.e., recently dead by other causes with an inactive GPS signal, an optical collar or an ear tag)

LD (i.e., long dead).

Matrix with departure states (t) in rows and arrival states (t^+) in columns are used to represent multievent models. The initial state probabilities (vector 1) correspond to the proportions of newly marked animals belonging to each considered state (α= active GPS collar, β=optical collar, 1- α- β = ear tag). Here, the initial state was known with certitude for every captured individual and this parameter is not necessary to be

estimated (we only modelled transition and event probabilities). Note that our model only includes uncertainty for dead individuals and animals cannot be captured and marked for the first time as dead.

$$\begin{matrix} A_{AG} & A_{OC} & A_{ET} & A_{FG} & DP_{G} & DP_{CT} & DO_{G} & DO_{CT} \end{matrix}$$
$$\begin{pmatrix} \alpha & \beta & 1-\alpha-\beta & 0 & 0 & 0 & 0 & 0 \end{pmatrix} \quad \text{Vector 1}$$

We broke down the transition between the state probabilities into two steps: the first step corresponds to the probabilities of losing the GPS signal (Ψ) and the entire GPS collar (σ). Probabilities of losing the GPS signal were modeled as a function of GPS age (i.e., time elapsed since the animal was equipped with the GPS collar). Note that we also modeled in this step the probability of permanent transition from recently dead states to a long dead state.

$$\begin{array}{c|ccccccccc} & A_{AG} & A_{OC} & A_{ET} & A_{FG} & DP_{G} & DP_{CT} & DO_{G} & DO_{CT} & LD \\ A_{AG} & 1-\psi-\sigma & 0 & \sigma & \psi & 0 & 0 & 0 & 0 & 0 \\ A_{OC} & 0 & 1 & 0 & 0 & 0 & 0 & 0 & 0 & 0 \\ A_{ET} & 0 & 0 & 1 & 0 & 0 & 0 & 0 & 0 & 0 \\ A_{FG} & 0 & 0 & \sigma & 1-\sigma & 0 & 0 & 0 & 0 & 0 \\ DP_{G} & 0 & 0 & 0 & 0 & 0 & 0 & 0 & 0 & 1 \\ DP_{CT} & 0 & 0 & 0 & 0 & 0 & 0 & 0 & 0 & 1 \\ DO_{G} & 0 & 0 & 0 & 0 & 0 & 0 & 0 & 0 & 1 \\ DO_{CT} & 0 & 0 & 0 & 0 & 0 & 0 & 0 & 0 & 1 \\ LD & 0 & 0 & 0 & 0 & 0 & 0 & 0 & 0 & 1 \end{array} \quad \text{Matrix 1}$$

The second step corresponds to the probability mortality due to poaching (η_p), due to other causes (η_o) and the complementary survival probability (ϕ=1-η_p-η_o) (matrix 2). Mortality probabilities were modeled as a function of individual age (following

different age structures) and considering the different hypotheses of interest (see main text).

$$
\begin{array}{c} \\ A_{AG} \\ A_{OC} \\ A_{ET} \\ A_{FG} \\ DP_{G} \\ DP_{CT} \\ DO_{G} \\ DO_{CT} \\ LD \end{array}
\begin{array}{c}
\begin{array}{ccccccccc} A_{AG} & A_{OC} & A_{ET} & A_{FG} & DP_{G} & DP_{CT} & DO_{G} & DO_{CT} & LD \end{array} \\
\begin{pmatrix}
\phi & 0 & 0 & 0 & \eta_p & 0 & \eta_o & 0 & 0 \\
0 & \phi & 0 & 0 & 0 & \eta_p & 0 & \eta_o & 0 \\
0 & 0 & \phi & 0 & 0 & \eta_p & 0 & \eta_o & 0 \\
0 & 0 & 0 & \phi & 0 & \eta_p & 0 & \eta_o & 0 \\
0 & 0 & 0 & 0 & 1 & 0 & 0 & 0 & 0 \\
0 & 0 & 0 & 0 & 0 & 1 & 0 & 0 & 0 \\
0 & 0 & 0 & 0 & 0 & 0 & 1 & 0 & 0 \\
0 & 0 & 0 & 0 & 0 & 0 & 0 & 1 & 0 \\
0 & 0 & 0 & 0 & 0 & 0 & 0 & 0 & 1
\end{pmatrix}
\end{array}
\quad \text{Matrix 2}
$$

The event probabilities were decomposed in two steps. First step (matrix 3) corresponds to the resighting probabilities (*p*) of animals marked with different methods (p_c=optical collar and inactive GPS collar; p_{et}=ear tag); and the recovery probability (*r*) of animals with optical collars, inactive GPS collars or ear tags. Both resighting and recovery were allowed to vary with time (due to potential temporal differences in monitoring effort). Detection of animals with active GPS collar was modelled as perfect (probability of 1). Note that in this step only 5 events (‘0’ to ‘4’) are reached from the states of departure. As uncertainty in the cause of mortality exist, the final events are reached from the recently dead states in the next step.

$$
\begin{array}{c|ccccccccc|}
 & '0' & '1' & '2' & '3' & '4' & DP_G & DP_{CT} & DO_G & DO_{CT} \\
A_{AG} & 0 & 1 & 0 & 0 & 0 & 0 & 0 & 0 & 0 \\
A_{OC} & 1-p_c & 0 & p_c & 0 & 0 & 0 & 0 & 0 & 0 \\
A_{ET} & 1-p_{et} & 0 & 0 & p_{et} & 0 & 0 & 0 & 0 & 0 \\
A_{FG} & 1-p_c & 0 & 0 & 0 & p_c & 0 & 0 & 0 & 0 \\
DP_G & 0 & 0 & 0 & 0 & 0 & 1 & 0 & 0 & 0 \\
DP_{CT} & 1-r & 0 & 0 & 0 & 0 & 0 & r & 0 & 0 \\
DO_G & 0 & 0 & 0 & 0 & 0 & 0 & 0 & 1 & 0 \\
DO_{CT} & 1-r & 0 & 0 & 0 & 0 & 0 & 0 & 0 & r \\
LD & 1 & 0 & 0 & 0 & 0 & 0 & 0 & 0 & 0
\end{array}
\quad \text{Matrix 3}
$$

Second step (matrix 4) corresponds to the probabilities of cause of death determination (δ) for recently dead individuals. This parameter, that accounts for uncertainty, is modeled as constant.

$$
\begin{array}{c|ccccccccccc|}
 & '0' & '1' & '2' & '3' & '4' & '5' & '6' & '7' & '8' & '9' & '10' \\
'0' & 1 & 0 & 0 & 0 & 0 & 0 & 0 & 0 & 0 & 0 & 0 \\
'1' & 0 & 1 & 0 & 0 & 0 & 0 & 0 & 0 & 0 & 0 & 0 \\
'2' & 0 & 0 & 1 & 0 & 0 & 0 & 0 & 0 & 0 & 0 & 0 \\
'3' & 0 & 0 & 0 & 1 & 0 & 0 & 0 & 0 & 0 & 0 & 0 \\
'4' & 0 & 0 & 0 & 0 & 1 & 0 & 0 & 0 & 0 & 0 & 0 \\
DP_G & 0 & 0 & 0 & 0 & 0 & \delta & 0 & 0 & 0 & 1-\delta & 0 \\
DP_{CT} & 0 & 0 & 0 & 0 & 0 & 0 & \delta & 0 & 0 & 0 & 1-\delta \\
DO_G & 0 & 0 & 0 & 0 & 0 & 0 & 0 & \delta & 0 & 1-\delta & 0 \\
DO_{CT} & 0 & 0 & 0 & 0 & 0 & 0 & 0 & 0 & \delta & 0 & 1-\delta
\end{array}
\quad \text{Matrix 4}
$$
