# Supplementary material for: Unravelling the sex- and age-specific impact of poaching mortality with multievent modeling
Source: Front Zool. 2019 Jun 13;16:20. doi: 10.1186/s12983-019-0321-1 (PMC6567384; doi:10.1186/s12983-019-0321-1)
Supplement: Supplementary file 2 — Multievent model representation. (PDF 163 kb) [file 12983_2019_321_MOESM2_ESM.pdf]

Additional file 2

Multievent model representation

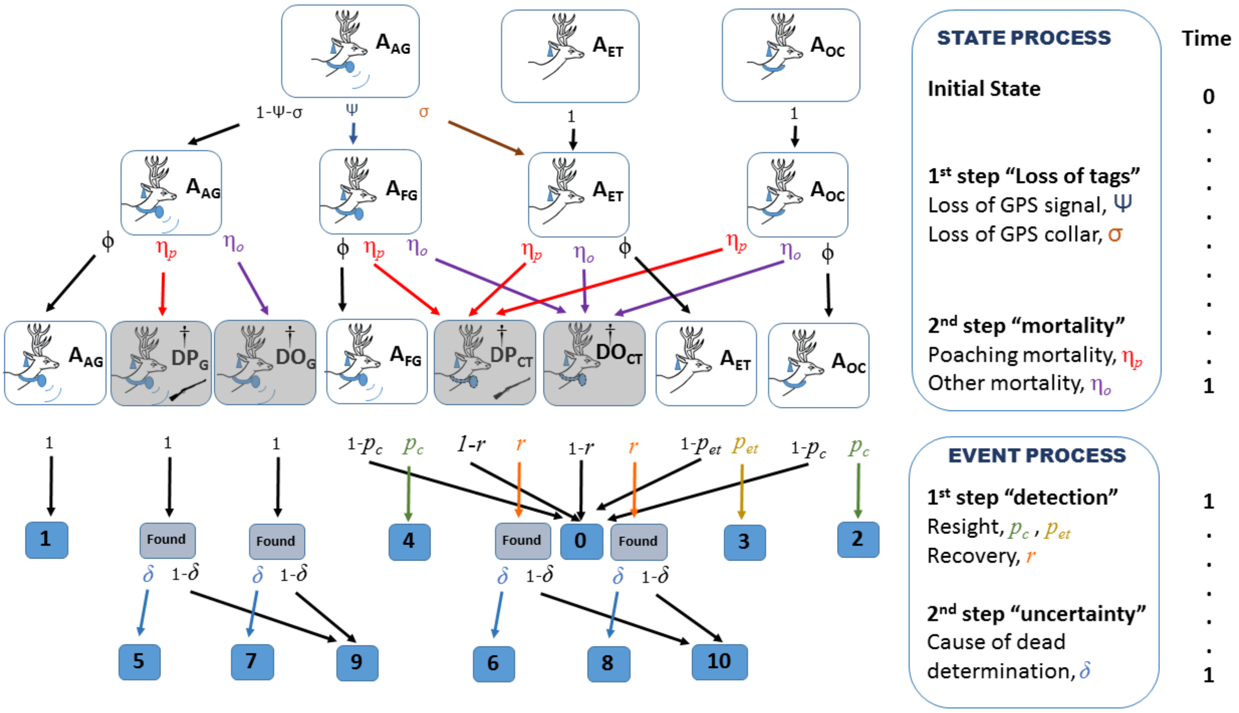

Figure 1. Schematic representation of multievent model (Initial state, state process and event process) between time 0 and time 1. The parameters estimated at each step are noted with different colors (their complementary parameters are in black). Alive states are noted by white boxes and dead states by grey boxes. The different events observed for each individual state are in blue boxes. Note that the mandatory transition (i.e., probability =1) between the recently dead states and the long dead state (occurring between time 1 and time 2 onwards) is not shown. Similarly, the probability of GPS collar lost for individuals without active GPS signal (i.e., from state A_{FG} to state A_{ET}) only occurring from between time 1 and time 2 onwards is not shown for simplicity.
